# Supplementary material for: Federated analysis of BRCA1 and BRCA2 variation in a Japanese cohort
Source: Cell Genom. 2022 Mar 9;2(3):100109. doi: 10.1016/j.xgen.2022.100109 (PMC8975122; doi:10.1016/j.xgen.2022.100109)
Supplement: Document S1. Methods S1–S3 [file mmc1.pdf]

Cell Genomics, Volume *2*

Supplemental information

Federated analysis of BRCA1 and BRCA2 variation in a Japanese cohort

James Casaletto, Michael Parsons, Charles Markello, Yusuke Iwasaki, Yukihide Momozawa, Amanda B. Spurdle, and Melissa Cline

Supplemental Information

There are 3 independent reports generated from our software solution: the variant co-occurrence and allele frequency report, the optional data quality report, and the genotype-phenotype intersection report. This section defines the detailed configuration for and output of each of these reports with an associated example.

The optional data quality report, discussed in the Design section and the Method Details section of the main text, supports multiple types of field values, including categorical, numerical, and “free-form”. The report also validates field values if defined in the `fieldValues` list. Any values found in the pathology data file not in this list are flagged in the report. If this list is left empty, then the report doesn’t perform this validation step. In our research, we used the data quality report to make sure that the data values fell within an acceptable range.

The optional genotype-phenotype intersection report is discussed in the Design section and Method Details section of the main text. If the optional phenotype data file is provided as a tab-separated file whose ‘ID’ fields match those used in the VCF file, then our software will intersect the VUS with the phenotype data. The intersection reveals which phenotypes are associated with which genomic variation.

The mandatory co-occurrence and allele frequency report, described in the Design section and Method Details section of the main text, provides the cohort frequency as well as the population and frequency of the population with the highest allele frequency. This report distinguishes between homozygous and heterozygous co-occurrences, and in the case of heterozygous ones, the report lists the known pathogenic variants which co-occur with the VUS.

We show 3 examples of using our software: one with associated phenotype data on BRCA2, one without associated phenotype data on BRCA1, and one with associated phenotype data on MYH7.

Methods S1: Generating reports using BRCA2 variants with associated phenotype (pathology) data, Related to STAR Methods

The following is a basic example which we will use to generate a data quality report, a phenotype-genotype intersection report, and an allele frequency and co-occurrence report. These reports are discussed in detail in the Design section and Method Details section of the main text.

1. Examine the `brca2.vcf` VCF file in the `examples/BRCA/data` directory.

```
##fileformat=VCFv4.2
#CHROM  POS       ID   REF     ALT   QUAL  FILTER  INFO  FORMAT  01    02    03    04
chr13   32355250  .    T       C     .     .       .     GT      1|0   0|0   0|0   0|0
chr13   32316508  .    GAC     G     .     .       .     GT      0|1   0|0   0|0   0|0
chr13   32353470  .    A       C     .     .       .     GT      0|0   1|0   0|0   0|0
chr13   32340836  .    GACAA   G     .     .       .     GT      0|0   0|1   0|0   0|0
chr13   32353519  .    A       G     .     .       .     GT      0|0   0|0   1|0   0|0
chr13   32338749  .    AATTAC  A     .     .       .     GT      0|0   0|0   0|1   0|0
chr13   32355250  .    T       C     .     .       .     GT      0|0   0|0   0|0   1|1
```

2. Examine the associated phenotype file `brca2-pathology.tsv` in the `examples/BRCA/data` directory.

```
ID      Family history / breast cancer  Age at onset    ER      PgR     HER2    CarrierGene
01      1       57      Positive        Positive        3+      BRCA2
02      0       51      Negative        Positive        1+      BRCA2
03      0       66      Negative        Negative        Negative        BRCA2
04      0       0       NA      NA      NA      NonCarrier
```

3. Examine the BRCA2 variant pathogenicity file `clinvar_brca2.tsv` in the `examples/BRCA/data` directory.

```
Clinical_significance   Genomic_Coordinate_hg37 Genomic_Coordinate_hg38
Pathogenic      -       chr13:32316508:GAC>G
Pathogenic      -       chr13:32340836:GACAA>G
Pathogenic      -       chr13:32338749:AATTAC>A
-       -       chr13:32355250:T>C
-       -       chr13:32355250:T>C
-       -       chr13:32353470:A>C
-       -       chr13:32353519:A>G
```

4. Examine the data quality report `config/brca2-report-config.json` JSON configuration file.

```
{
  "qualityReport": "data/BRCA2-data-quality-report.txt",
  "pathologyReport": "/dev/null",
  "fileName": "data/brca2-pathology.tsv",
  "fileHeader": "True",
  "fieldDelimiter": "\t",
  "printConfigFileInfo": "False",
  "printBadValues": "True",
  "suppressAllOutput": "False",
  "RScriptPath": "/usr/bin/Rscript",
  "fieldFilters": [
    {"fieldName": "ER", "fieldType": "categorical",
        "fieldValues": ["Positive", "Negative", "NA"], "printFieldCount": "True"},
    {"fieldName": "PgR", "fieldType": "categorical",
        "fieldValues": ["Positive", "Negative", "NA"], "printFieldCount": "True"},
    {"fieldName": "HER2", "fieldType": "categorical",
        "fieldValues": ["0", "1+", "2+", "3+", "NA"], "printFieldCount": "True"},
    {"fieldName": "Age at onset", "fieldType": "numerical",
        "fieldValues": {"low": 10, "high": 150}, "printFieldCount": "True", "printStats": "True"},
    {"fieldName": "Family history / breast cancer", "fieldType": "numerical",
        "fieldValues": [0, 1], "printFieldCount": "True", "printStats": "True"},
    {"fieldName": "CarrierGene", "fieldType": "categorical",
        "fieldValues": [], "printFieldCount": "True"}]
}
```

5. Examine the `gnomad_chr13_brca2.tsv` file in the `examples/BRCA/data` directory. Note the screenshot was truncated as the contents of the file are too large to display here.

```
chr13   32316508        rs80359483      GAC     G       .       PASS    AC=1;AN=152032;AF=6.57756e-06;popma
x=nfe;faf95_popmax=0
```

6. Run the report workflow.

```
./runMe.sh -c 13 \
-p True \
-g BRCA2 \
-dd $(pwd)/examples/BRCA/data \
-cd $(pwd)/examples/BRCA/config \
-vf brca2.vcf \
-vpf clinvar_brca2.tsv \
-rc brca2-report-config.json \
-spf brca2-pathology.tsv \
-gf gnomad_chr13_brca2.vcf
```

   Here `-c` is the chromosome number, `-p` is whether the data is phased, `-g` is the name of the gene, `-dd` is the absolute path to the data directory, `-cd` is the absolute path to the configuration directory, `-vf` is the name of the VCF file, `-vpf` is the name of the pathogenicity file in the `data` directory, `-rc` is the name of the report configuration file in the `config` directory, `-spf` is the name of the sample phenotype file in the `data` directory, and `-gf` is the name of the gnomad allele frequency file in the `data` directory.

7. Examine the co-occurrence and allele frequency report.

```
{
    "cooccurring vus": {
        "(13, 32355250, 'T', 'C')": {
            "likelihood data": { "p1": 0.375, "p2": 0.001, "n": 2, "k": 1,
                "likelihood": 0.0042624 },
            "allele frequencies": { "maxPop": null, "maxPopFreq": 0.0,
                "minPop": null, "minPopFreq": 1.0, "cohortFreq": 0.5 },
            "pathogenic variants": [ [ 13, 32316508, "GAC", "G" ] ]
        },
        "(13, 32353470, 'A', 'C')": {
            "likelihood data": { "p1": 0.375, "p2": 0.001, "n": 1, "k": 1,
                "likelihood": 0.0026666666666666666 },
            "allele frequencies": { "maxPop": null, "maxPopFreq": 0.0,
                "minPop": null, "minPopFreq": 1.0, "cohortFreq": 0.25 },
            "pathogenic variants": [ [ 13, 32340836, "GACAA", "G" ] ]
        },
        "(13, 32353519, 'A', 'G')": {
            "likelihood data": { "p1": 0.375, "p2": 0.001, "n": 1, "k": 1,
                "likelihood": 0.0026666666666666666 },
            "allele frequencies": { "maxPop": null, "maxPopFreq": 0.0,
                "minPop": null, "minPopFreq": 1.0, "cohortFreq": 0.25 },
            "pathogenic variants": [ [ 13, 32338749, "AATTAC", "A" ] ]
        }
    },
    "homozygous vus": {
        "(13, 32355250, 'T', 'C')": { "count": 1, "maxPop": null, "maxPopFreq": 0.0,
                "minPop": null, "minPopFreq": 1.0, "cohortFreq": 0.25 }
    }
}
```

8. Examine the data quality report.

```
==============================================
total records read from data file: 4
==============================================
column: ER / type: categorical
{
    "fieldCount": { "NA": 1, "Negative": 2, "Positive": 1 }
}
==============================================
column: PgR / type: categorical
{
    "fieldCount": { "NA": 1, "Negative": 1, "Positive": 2 }
}
==============================================
column: HER2 / type: categorical
{
    "fieldCount": { "1+": 1, "3+": 1, "NA": 1 }
}
==============================================
column: Age at onset / type: numerical
{
    "fieldCount": { "51": 1, "57": 1, "66": 1 }
}
min = 51, max = 66, mean = 58 median = 57 stdev = 7.54983443527075
==============================================
column: Family history / breast cancer / type: numerical
{
    "fieldCount": { "0": 3, "1": 1 }
}
min = 0, max = 1, mean = 0.25 median = 0.0 stdev = 0.5
==============================================
column: CarrierGene / type: categorical
{
    "fieldCount": { "BRCA2": 3, "NonCarrier": 1 }
}
==============================================
bad values: {2: [('HER2', 'Negative')], 3: [('Age at onset', 0)]}
==============================================
missing values: {}
```

9. Examine the phenotype-genotype intersection report.

```
{
    "cooccurring": {
        "(13, 32353470, 'A', 'C')": {
            "phenotype": [ { "Age at onset": [ 51 ], "CarrierGene": [ "BRCA2" ],
                        "ER": [ "Negative" ], "Family history / breast cancer": [ 0 ],
                        "HER2": [ "1+" ], "ID": [ 2 ], "PgR": [ "Positive" ] } ]
        },
        "(13, 32353519, 'A', 'G')": {
            "phenotype": [ { "Age at onset": [ 66 ], "CarrierGene": [ "BRCA2" ],
                        "ER": [ "Negative" ], "Family history / breast cancer": [ 0 ],
                        "HER2": [ "Negative" ], "ID": [ 3 ], "PgR": [ "Negative" ] } ]
        },
        "(13, 32355250, 'T', 'C')": {
            "phenotype": [ { "Age at onset": [ 57 ], "CarrierGene": [ "BRCA2" ],
                        "ER": [ "Positive" ], "Family history / breast cancer": [ 1 ],
                        "HER2": [ "3+" ], "ID": [ 1 ], "PgR": [ "Positive" ] } ]
        }
    },
    "homozygous": {
        "(13, 32355250, 'T', 'C')": {
            "phenotype": [ { "Age at onset": [ 0 ], "CarrierGene": [ "NonCarrier" ],
                        "ER": [ NaN ], "Family history / breast cancer": [ 0 ],
                        "HER2": [ NaN ], "ID": [ 4 ], "PgR": [ NaN ] } ] }
    },
    "numMissing": 0
}
```

Methods S2: Generating reports using BRCA1 variants without associated phenotype data, Related to STAR Methods

The following is a basic example which we will use to generate a co-occurrence and allele frequency report. These reports are discussed in detail in the Design section and Method Details section of the main text.

1. Examine the `brca1.vcf` VCF file in the `examples/BRCA/data` directory.

```
#CHROM  POS       ID    REF   ALT   QUAL  FILTER  INFO  FORMAT  01    02    03
17      43074403  .     C     A     .     .       .     GT      0|0   0|0   1|0
17      43088049  .     G     A     .     .       .     GT      1|0   0|1   1|1
17      43110245  .     T     G     .     .       .     GT      0|0   1|1   0|0
17      43124027  .     ACT   A     .     .       .     GT      0|1   1|0   0|0
```

2. Examine the BRCA2 variant pathogenicity file `brca2-pathogenicity.tsv` in the `examples/BRCA/data` directory.

```
Clinical_significance   Genomic_Coordinate_hg37 Genomic_Coordinate_hg38
Pathogenic      -       chr17:43074403:C>A
Pathogenic      -       chr17:43124027:ACT>A
-       -       chr17:43088049:G>A
-       -       chr17:43110245:T>G
```

3. Run the report workflow.

```
./runMe.sh -c 17 \
-p True \
-g BRCA1 \
-dd $(pwd)/examples/BRCA/data \
-cd $(pwd)/examples/BRCA/config \
-vf brca1.vcf \
-vpf clinvar_brca1.tsv \
-gf gnomad_chr17_brca1.vcf
```

4. Examine the co-occurrence and allele frequency report in the `data` directory.

```
{
    "cooccurring vus": {
        "(14, 23413860, 'G', 'A')": {
            "likelihood data": { "p1": 0.5, "p2": 0.001, "n": 2, "k": 2, "likelihood": 4e-06
            },
            "allele frequencies": { "maxPop": "nfe", "maxPopFreq": "4.88e-06", "cohortFreq": 0.5
            },
            "pathogenic variants": [ [ 14, 23413847, "T", "A" ] ]
        },
        "(14, 23413889, 'T', 'C')": {
            "likelihood data": { "p1": 0.5, "p2": 0.001, "n": 2, "k": 2, "likelihood": 4e-06
            },
            "allele frequencies": { "maxPop": "afr", "maxPopFreq": "1.919e-05", "cohortFreq": 0.5
            },
            "pathogenic variants": [ [ 14, 23413847, "T", "A" ], [ 14, 23414002, "C", "G" ] ]
        },
        "(14, 23413831, 'T', 'G')": {
            "likelihood data": { "p1": 0.5, "p2": 0.001, "n": 3, "k": 3, "likelihood": 8e-09
            },
            "allele frequencies": { "maxPop": "nfe", "maxPopFreq": "0.00018197", "cohortFreq": 0.75
            },
            "pathogenic variants": [ [ 14, 23414002, "C", "G" ], [ 14, 23413847, "T", "A" ] ]
        }
    },
    "homozygous vus": {
        "(14, 23413889, 'T', 'C')": { "count": 1, "maxPop": "afr", "maxPopFreq": "1.919e-05", "cohortFreq"
: 0.25
        },
        "(14, 23413831, 'T', 'G')": { "count": 1, "maxPop": "nfe", "maxPopFreq": "0.00018197", "cohortFreq
": 0.25
        }
    }
}
```

Methods S3: Generating reports using MYH7 variants with associated phenotype (cardiac) data, Related to STAR Methods

The following is a basic example which we will use to generate a data quality report, a co-occurrence and allele frequency report, and a cardiac report. Note that since we have an associated phenotype file, we will also generate a fourth report which intersects VUS with cardiac data. These reports are discussed in detail in the Design section and Method Details section of the main text.

1. Examine the `myh7.vcf` VCF file in the `examples/MYH7/data` directory.

```
##fileformat=VCFv4.2
#CHROM  POS       ID   REF  ALT  QUAL  FILTER  INFO  FORMAT  01   02   03   04
14      23413831  .    T    G    .     .       .     GT      0|0  1|0  0|1  1|1
14      23413860  .    G    A    .     .       .     GT      1|0  0|0  0|0  0|1
14      23413889  .    T    C    .     .       .     GT      1|1  0|0  1|0  0|0
14      23413847  .    T    A    .     .       .     GT      0|1  0|0  0|0  1|0
14      23414002  .    C    G    .     .       .     GT      1|0  0|1  1|0  0|0
```

2. Examine the associated phenotype file `myh7-cardiac.tsv` in the `examples/MYH7/data` directory.

```
ID    Age at onset    Native T1 (msec)    Technique    ECV
01    57              976                 MOLLI        25.9
02    51              1159                ShMOLLI      26.9
03    66              1122                MOLLI        26.4
04    0               994                 MOLLI        25.4
```

3. Examine the ClinVar pathogenicity file `gnomad_myh7.tsv` in the `examples/MYH7/data` directory.

```
Clinical_significance   Genomic_Coordinate_hg37 Genomic_Coordinate_hg38
Pathogenic      -       chr14:23413847:T>A
Pathogenic      -       chr14:23414002:C>G
Conflicting interpretations of pathogenicity    -       chr14:23413831:T>G
Uncertain significance  -       chr14:23413860:G>A
Uncertain significance  -       chr14:23413889:T>C
Likely pathogenic       -       chr14:23413890:CC>C
```

4. Examine the data quality report `config/myh7-report-config.json` JSON configuration file.

```
{
  "qualityReport": "data/MYH7-data-quality-report.txt",
  "pathologyReport": "data/MYH7-cardiac-report.txt",
  "fileName": "data/myh7-cardiac.tsv",
  "fileHeader": "True",
  "fieldDelimiter": "\t",
  "printConfigFileInfo": "False",
  "printBadValues": "False",
  "suppressAllOutput": "False",
  "RScriptPath": "/usr/bin/Rscript",
  "fieldFilters": [
    {"fieldName": "Technique", "fieldType": "categorical",
        "fieldValues": ["MOLLI", "ShMOLLI", "NA"], "printFieldCount": "True"},
    {"fieldName": "Age at onset", "fieldType": "numerical",
        "fieldValues": [], "printFieldCount": "True", "printStats": "True"},
    {"fieldName": "Native T1 (msec)", "fieldType": "numerical",
        "fieldValues": [], "printFieldCount": "True", "printStats": "True"},
    {"fieldName": "ECV", "fieldType": "numerical",
        "fieldValues": [], "printFieldCount": "True", "printStats": "True"}]
}
```

5. Examine the `gnomad_chr14_myh7.tsv` file in the `examples/MYH7/data` directory. Note the screenshot was truncated as the contents of the file are too large to display here.

```
chr14   23413831        rs45523233      T       G       .       PASS    AC=23;AN=152258;AF=0.000151059;popm
ax=nfe;faf95_popmax=0.00018197;
```

6. Run the report workflow.

```
./runMe.sh -c 14 \
-p True \
-g MYH7 \
-dd $(pwd)/examples/MYH7/data \
-cd $(pwd)/examples/MYH7/config \
-vf myh7.vcf \
-vpf clinvar_myh7.tsv \
-gf gnomad_chr14_myh7.vcf \
-rc myh7-report-config.json \
-spf myh7-cardiac.tsv
```

Here `-c` is the chromosome number, `-p` is whether the data is phased, `-g` is the name of the gene, `-dd` is the absolute path to the data directory, `-cd` is the absolute path to the configuration directory, `-vf` is the name of the VCF file in the `data` directory, `-vpf` is the name of the pathogenicity file in the `data` directory, `-gf` is the name of the gnomad allele frequency file in the `data` directory, `-rc` is the name of the report configuration file in the `config` directory, and `-spf` is the name of the phenotype file in the `data` directory

7. Examine the data quality report.

```
{
    "cooccurring vus": {
        "(14, 23413860, 'G', 'A')": {
            "likelihood data": { "p1": 0.5, "p2": 0.001, "n": 2, "k": 2, "likelihood": 4e-06
            },
            "allele frequencies": { "maxPop": "nfe", "maxPopFreq": "4.88e-06", "cohortFreq": 0.5
            },
            "pathogenic variants": [ [ 14, 23413847, "T", "A" ] ]
        },
        "(14, 23413889, 'T', 'C')": {
            "likelihood data": { "p1": 0.5, "p2": 0.001, "n": 2, "k": 2, "likelihood": 4e-06
            },
            "allele frequencies": { "maxPop": "afr", "maxPopFreq": "1.919e-05", "cohortFreq": 0.5
            },
            "pathogenic variants": [ [ 14, 23413847, "T", "A" ], [ 14, 23414002, "C", "G" ] ]
        },
        "(14, 23413831, 'T', 'G')": {
            "likelihood data": { "p1": 0.5, "p2": 0.001, "n": 3, "k": 3, "likelihood": 8e-09
            },
            "allele frequencies": { "maxPop": "nfe", "maxPopFreq": "0.00018197", "cohortFreq": 0.75
            },
            "pathogenic variants": [ [ 14, 23414002, "C", "G" ], [ 14, 23413847, "T", "A" ] ]
        }
    },
    "homozygous vus": {
        "(14, 23413889, 'T', 'C')": { "count": 1, "maxPop": "afr", "maxPopFreq": "1.919e-05", "cohortFreq"
: 0.25
        },
        "(14, 23413831, 'T', 'G')": { "count": 1, "maxPop": "nfe", "maxPopFreq": "0.00018197", "cohortFreq
": 0.25
        }
    }
}
```

8. Examine the co-occurrence and allele frequency report.

```
{
    "cooccurring vus": {
        "(14, 23413860, 'G', 'A')": {
            "likelihood data": { "p1": 0.5, "p2": 0.001, "n": 2, "k": 2, "likelihood": 4e-06
            },
            "allele frequencies": { "maxPop": "nfe", "maxPopFreq": "4.88e-06", "cohortFreq": 0.5
            },
            "pathogenic variants": [ [ 14, 23413847, "T", "A" ] ]
        },
        "(14, 23413889, 'T', 'C')": {
            "likelihood data": { "p1": 0.5, "p2": 0.001, "n": 2, "k": 2, "likelihood": 4e-06
            },
            "allele frequencies": { "maxPop": "afr", "maxPopFreq": "1.919e-05", "cohortFreq": 0.5
            },
            "pathogenic variants": [ [ 14, 23413847, "T", "A" ], [ 14, 23414002, "C", "G" ] ]
        },
        "(14, 23413831, 'T', 'G')": {
            "likelihood data": { "p1": 0.5, "p2": 0.001, "n": 3, "k": 3, "likelihood": 8e-09
            },
            "allele frequencies": { "maxPop": "nfe", "maxPopFreq": "0.00018197", "cohortFreq": 0.75
            },
            "pathogenic variants": [ [ 14, 23414002, "C", "G" ], [ 14, 23413847, "T", "A" ] ]
        }
    },
    "homozygous vus": {
        "(14, 23413889, 'T', 'C')": { "count": 1, "maxPop": "afr", "maxPopFreq": "1.919e-05", "cohortFreq"
: 0.25
        },
        "(14, 23413831, 'T', 'G')": { "count": 1, "maxPop": "nfe", "maxPopFreq": "0.00018197", "cohortFreq
": 0.25
        }
    }
}
```

9. Examine the phenotype-genotype intersection report.

```
{
    "cooccurring": {
        "(14, 23413831, 'T', 'G')": {
            "phenotype": [
                { "Age at onset": [ 66 ], "ECV": [ 26.4 ], "Native T1 (msec)": [ 1122 ],
                    "Technique": [ "MOLLI" ] },
                { "Age at onset": [ 51 ], "ECV": [ 26.9 ], "Native T1 (msec)": [ 1159 ],
                    "Technique": [ "ShMOLLI" ] } ] },
        "(14, 23413860, 'G', 'A')": {
            "phenotype": [
                { "Age at onset": [ 57 ], "ECV": [ 25.9 ], "Native T1 (msec)": [ 976 ],
                    "Technique": [ "MOLLI" ] },
                { "Age at onset": [ 0 ], "ECV": [ 25.4 ], "Native T1 (msec)": [ 994 ],
                    "Technique": [ "MOLLI" ] } ] },
        "(14, 23413889, 'T', 'C')": {
            "phenotype": [
                { "Age at onset": [ 66 ], "ECV": [ 26.4 ], "Native T1 (msec)": [ 1122 ],
                    "Technique": [ "MOLLI" ] } ] } },
    "homozygous": {
        "(14, 23413831, 'T', 'G')": {
            "phenotype": [
                { "Age at onset": [ 0 ], "ECV": [ 25.4 ], "Native T1 (msec)": [ 994 ],
                    "Technique": [ "MOLLI" ] } ] },
        "(14, 23413889, 'T', 'C')": {
            "phenotype": [
                { "Age at onset": [ 57 ], "ECV": [ 25.9 ], "Native T1 (msec)": [ 976 ],
                    "Technique": [ "MOLLI" ] } ] } },
    "numMissing": 0
}
```
